# Effect of distributing urine-collection bags on contrast-material load in wastewater

**DOI:** 10.1007/s00330-025-11984-5

**Published:** 2025-10-17

**Authors:** Ben J. A. Janssen, Frank M. Zijta, Natasja Fraters, Ad de Man, Sandra Malagon, Saba Rafi, Henrius P. Raat, Ankie Hersbach, Joachim E. Wildberger, Estelle C. Nijssen

**Affiliations:** 1 Department of Pharmacology & Toxicology, Faculty of Health Medicine and Life Sciences (FHML), Maastricht University, Maastricht, The Netherlands; 2 Department of Radiology and Nuclear Medicine, Maastricht University Medical Centre+, Maastricht, The Netherlands; 3 Cardiovascular Research Institute Maastricht (CARIM), Maastricht University, Maastricht, The Netherlands; 4 Care and Public Health Research Institute (CAPHRI), Maastricht University, Maastricht, The Netherlands; 5 Water Board Limburg (Waterschap Limburg), Roermond, The Netherlands; 6 Department of Radiology, Laurentius Hospital Roermond, Roermond, The Netherlands

**Keywords:** Contrast media, Computed tomography, Magnetic resonance imaging, Environmental pollutants

## Abstract

**Objectives:**

Contrast materials (CM) are ubiquitous in modern clinical practice. Metabolically inert and excreted in urine, treatment plants (WWTP) have difficulty removing CM from wastewater and CM increasingly emerge as environmental contaminants. This study evaluates the effect of urine-collection bag (UCB) distribution on corresponding CM load in WWTP influent.

**Materials and methods:**

This prospective observational multicenter study includes patients scheduled for contrast-material-enhanced computed tomography (iodine-CM) or magnetic resonance imaging (gadolinium-CM) at an academic and a regional hospital. At each centre, data were collected over a 3-week control-period and a 3-week intervention-period with standard-clinical-care UCB distribution (4pp). Control and intervention were compared for cumulative iodine- and gadolinium-CM-loads in WWTP influent using linear regression analysis, corrected for administered CM. Compliance was evaluated in interviews with consenting patients; results were used to estimate achievable UCB-distribution effects.

**Results:**

UCB were distributed to 69.1% (1188/1719) eligible patients, and had a statistically significant effect on WWTP influent CM-loads: intervention versus control −17.4% iopromide [F(1,37) = 54.7, *p* < 0.001, *η*^2^ = 0.60; *R*^2^ = 0.966]; −14.8% ioversol [F(1,37) = 154.5, *p* < 0.001, *η*^2^ = 0.82; *R*^2^ = 0.989]; −7.2% gadolinium at the academic hospital [F(1,37) = 43.3, *p* < 0.001, *η*^2^ = 0.54, *R*^2^ = 0.967]; −33.2% gadolinium at the regional hospital [F(1,37) = 1.13, *p* = 0.296, *η*^2^ = 0.03]. Interviews were conducted with 47.0% (558/1188) patients: 92.1% (514/558) reported using UCB, and they used 89.2% (1834/2056) of the UCB they were provided with. Compliance-based estimates were: achievable compliance 29.9% to 43.6%, interceptable CM 26.7% to 38.9%.

**Conclusion:**

UCB distribution had a significant but small impact on reducing wastewater CM-loads. Compliance data overestimate UCB-distribution effect, which underscores the importance of wastewater measurements when evaluating mitigation strategies.

**Key Points:**

***Question*** *Contrast materials (CM) increasingly emerge as environmental contaminants; because treatment plants are currently unable to remove CM from wastewater, the effects of urine-collection-bag distribution are evaluated*.

***Findings*** *Standard-care urine-collection-bag distribution after CT and MRI led to small but significant CM-load reductions in wastewater; compliance data, however, led to sizeable overestimation of (achievable) effects*.

***Clinical relevance*** *Urine-collection bag distribution had a significant but small impact on reducing contrast materials in wastewater. Most studies only include compliance data, but results show these overestimate impact, underscoring the importance of contrast-load measurements when evaluating mitigation strategies*.

**Graphical Abstract:**

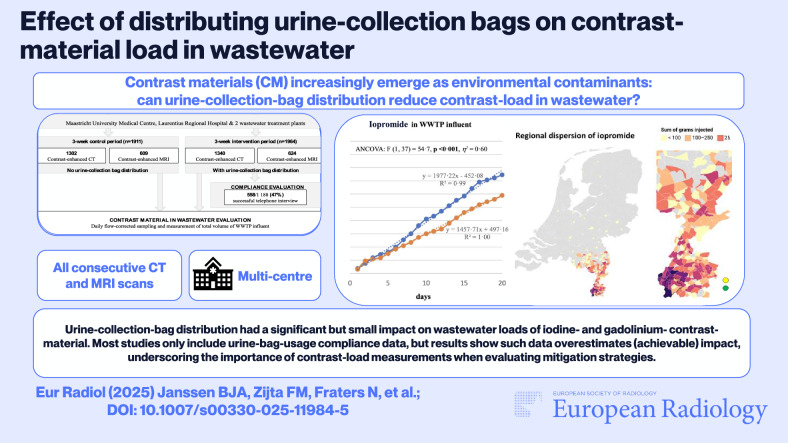

## Introduction

Millions of medical procedures use intravenous contrast materials (CM) to enhance diagnoses and treatment precision [1]. The most commonly administered CM are iodine-based CM for computed tomography (CT) scans, and gadolinium-based CM for magnetic resonance imaging (MRI). Modern CM are metabolically inactive and are typically excreted by renal filtration within 24 h after intravenous injection [2]. However, this same molecular stability hinders removal by wastewater treatment plants (WWTPs) [3, 4]. As a result, CM increasingly emerge as contaminants in environmental aquatic systems [5–8], posing ecotoxicological risks and introducing concerns about drinking water quality [9, 10].

Although circular gadolinium-based CM are quite stable, long-term data on environmental stability and effect are lacking [3]. In its free form, gadolinium has a negative impact on aquatic species in both freshwater and marine systems [11, 12]. Furthermore, depositions of gadolinium have been reported in various animals as well as in human (children and adult) tissues [13]. Iodine-based CM are also relatively stable [14], but transformation products, especially those derived from contact with other halogens such as chlorine and bromine, may lead to genotoxic products [4, 15].

The United Nations published a strategic approach in 2015 to motivate its members to take measures that will reduce the discharge of pharmaceuticals into the aquatic environment (Sustainable Development Goal 6: “Clean water and sanitation for all”) [16]. While legal frameworks to determine upper acceptable thresholds for contrast material contamination have not yet been established, many healthcare institutions are committed to mitigating initiatives such as minimizing CM emission into wastewater [6, 17–19].

Specific filters to remove CM from wastewater require dedicated sewage infrastructures. A more widely applicable measure is the distribution of urine-collection bags (UCB). The idea is that urine is collected over a 24-h period after contrast material-enhanced scans to intercept CM before it reaches wastewater [2, 20]. These UCB contain an absorbent gel and can be disposed of via non-recyclable waste systems. Incineration then destroys CM organic structure, leaving free iodine and gadolinium to end up in landfill material with reduced environmental impact [21]. The seeming simplicity and feasibility of this measure entice healthcare centers to consider routine UCB distribution. However, the (potentially achievable) effects of this measure in reducing wastewater CM load are unknown.

The current multicenter study informs on the achievable effects of UCB distribution. Control periods (without UCB distribution) and intervention periods (with UCB distribution) are compared for cumulative (iodine- and gadolinium-based) CM load in WWTP influence, based on continuous CM-load monitoring at WWTPs, corrected for amounts of intravenously administered CM. Participating centers were a regional and an academic hospital. In both cases, only standard-care hospital logistics (i.e., the standard daily management, planning and coordination within each healthcare facility), and personnel available for standard clinical care were used for UCB distribution. This was done to evaluate the feasibility of implementation in standard clinical care. UCB-distribution success, patient compliance, and patient provenance were evaluated.

## Materials and methods

All patients received standard care. Informed consent was obtained from participants for a follow-up telephone interview regarding UCB use. Ethical approval was obtained from the Maastricht Medical Ethics Committee (METC azM/UM 2022-3265).

### Study design

**Fig. 1:**
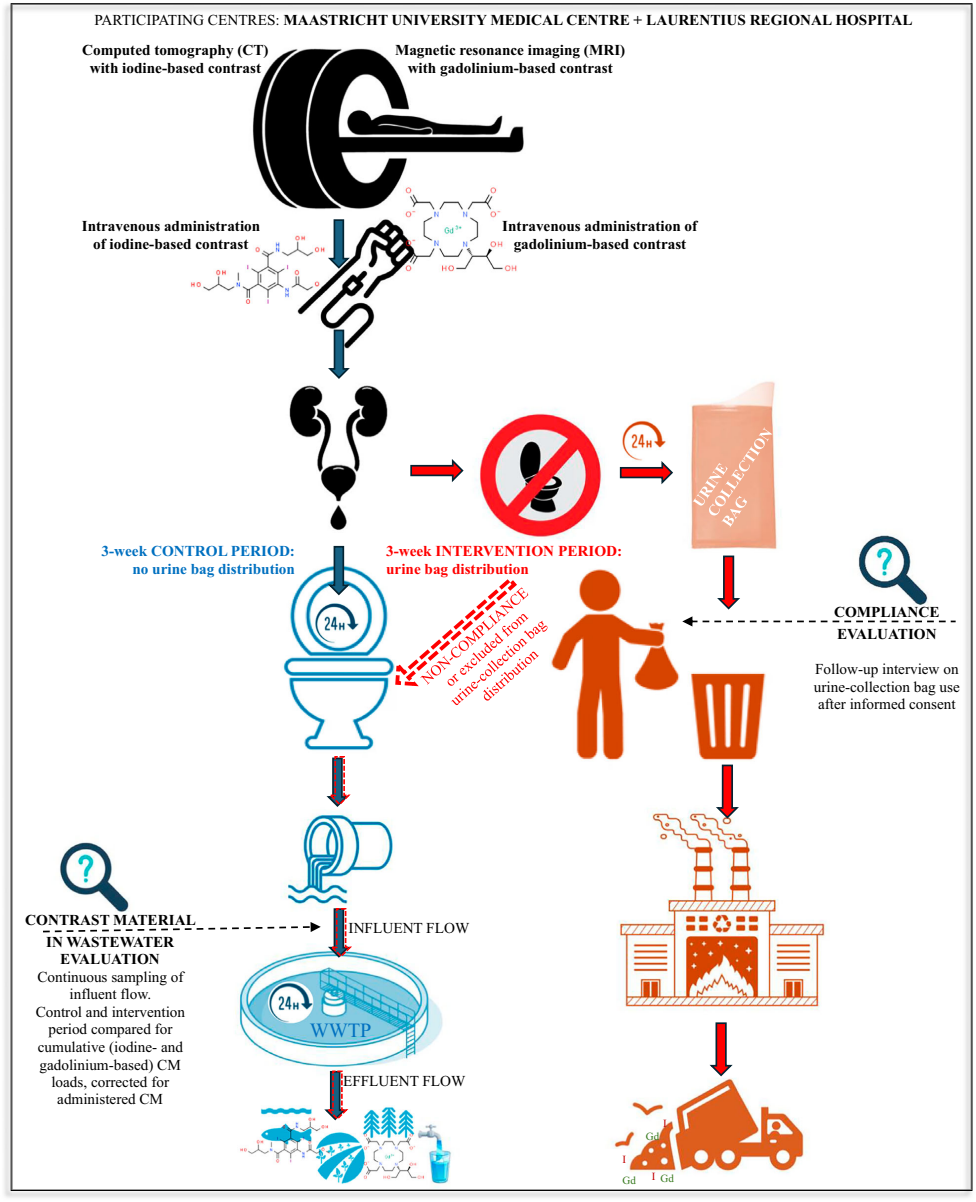
From injection to environment: contrast material route, including current study design. WWTP, wastewater treatment plant

This is a prospective observational multicenter study involving patients scheduled for contrast material enhanced CT or MRI at Maastricht University Medical Centre (academic hospital providing local primary to (inter-) national tertiary care at Maastricht, the Netherlands; hereafter referred to as “Maastricht UMC+”), and Laurentius Regional Hospital (Roermond, the Netherlands; hereafter referred to as “Laurentius RH”). Figure 1 gives an overview of the CM route from administration to environment, including the current study design.

All consecutive patients referred for contrast material-enhanced CT or MRI scans were eligible for inclusion; at Maastricht UMC+, hospitalized patients were excluded for logistic reasons. There were no other exclusion criteria.

Because data on clinically or environmentally relevant CM loads and effects of UCB distribution on CM loads are lacking, and as this is the first study of continuous CM-load monitoring at WWTPs, a formal power calculation could not be performed and study duration was based on feasibility.

Data were collected over a 3-week control period (without UCB distribution), and a 3-week intervention period (with UCB distribution to each consenting patient directly after completion of the scan). Periods without holidays were chosen to ensure normal 5-workday weeks during study periods. At Maastricht UMC+, the control period was between November 23 and December 12, 2022, and the intervention period was between January 18 and February 6, 2023. At Laurentius RH, the control period was between March 15 and April 2, 2024, and the intervention period was between February 23 and March 12, 2024.

### Participating centers

Characteristics of participating centers and local WWTP are given in Table 1. Besides expected differences in size between academic and regional hospital, main differences pertain to populations residing within the same WWTP catchment area as the hospital (Maastricht UMC+ 43,000 people versus Laurentius RH 120,000 people), and average dry-weather flow of local WWTP (Maastricht UMC+ 13,200 m^3^/day versus Laurentius RH 37,200 m^3^/day).

**Table 1:** Characteristics of participating medical centers and local wastewater treatment plants

	Maastricht University Medical Centre	Laurentius Regional Hospital
Level of healthcare provided	Local primary to (inter)national tertiary	Local secondary
Hospital capacity	715 beds	290 beds
Standard iodine-based contrast material	Iopromide 300 mgI/mL (Ultravist®)	Ioversol 350 mgI/mL (Optiray®)
Standard gadolinium-based contrast material	Gadobutrol 1.0 mmol Gd/mL (Gadovist®)	Gadoteric acid 0.5 mmol Gd/mL (Dotarem®)
WWTP receiving hospital effluents	Heugem	Roermond
Average dry-weather flow	13,200 m^3^/day	37,200 m^3^/day
Population residing within the catchment area	43,000 people	120,000 people
Automatic flow-corrected continuous sampling	50 mL/100m^3^	50 mL/300m^3^
Number of samples every 24 h at dry-weather flow	127	132

*CT* computed tomography, *MRI* magnetic resonance imaging, *I* iodine, *Gd* gadolinium

At Maastricht UMC+, contrast material-enhanced CT scans are done using iopromide (Ultravist^®^, Bayer Healthcare); at Laurentius RH, using ioversol (Optiray^®^, Guerbet). Contrast material-enhanced MRI scans are done using gadobutrol at Maastricht UMC+ (Gadovist^®^, Bayer), and using gadoteric acid at Laurentius RH (Dotarem^®^, Guerbet; Table 1).

### Urine-collection bag distribution and informed consent

Only logistics and personnel already present for standard clinical practice were used for UCB distribution. The head researcher, B.J., actively approached and instructed radiographer team leaders at both centers. Radiographers were informed of their tasks by their team leaders during regular work meetings.

General information for patients was provided via posters and screens in waiting rooms. In addition, radiographers were tasked with actively informing all contrast material-enhanced CT and MRI patients about the UCB, which was done during scan preparation, as well as with distributing UCB and instructing patients on UCB use, which was done after completion of the scan. Patients were instructed to start using UCB at the hospital if needed, and were each given a set of four UCB (HY-SUPP-URI4-000, DispoCare), based on an estimated requirement of bags per patient [2, 22].

Radiographers informed patients about the study and asked consenting patients to deposit the completed informed consent (IC) forms in a dedicated collection box in the waiting room (IC forms and collection boxes were placed in 5 waiting rooms at Maastricht UMC+, and in 1 waiting room at Laurentius RH).

Consenting patients were contacted by phone within 72 h after CM administration by one of the Health Medicine and Life Sciences students involved in the study (supervised by B.J.; see Acknowledgements). Up to two additional attempts to contact patients were made within the predefined timeframe, if necessary. Interviews were not fully structured but always included the following standard questions: “Did you use the urine-collection bags?”, “How many bags did you use?”, “Where did you use UCB (hospital, work, home)?”, and “Would you be prepared to use UCB in future?”. Reasons for not using UCB were often given during interviews, but are beyond the scope of this study.

### Outcomes

For the primary outcome, control and intervention periods are compared for cumulative iodine- and gadolinium-based CM loads in WWTP influent, corrected for administered CM.

The secondary outcome is UCB compliance, defined as the percentage of patients who reported using at least one UCB. In addition, how many UCB were used by each patient, where the UCB were used (hospital, work, home), and willingness to use UCB in future, were inventoried. Finally, UCB-distribution success during standard clinical care was evaluated, defined as the number of eligible patients who actually received UCB.

In order to gain insight into the number of patients residing within the local WWTP catchment area and thus into CM dispersion, hospitalized patients and provenance of outpatients were registered during control and intervention periods.

Results on distribution rates and compliance were used to estimate the achievable effects of UCB distribution:

- Minimum achievable compliance (proportion of patients eligible for UCB distribution who confirmed using UCB during the follow-up interview)
- Minimum interceptable CM (minimum achievable compliance multiplied by the proportion of UCB used by interviewed and complying patients)
- Maximum achievable compliance (proportion of patients consenting to follow-up, irrespective of whether they were actually interviewed, multiplied by known compliance of interviewed patients)
- Maximum interceptable CM (maximum achievable compliance multiplied by the proportion of UCB used by interviewed and complying patients).

Observed distribution success was the proportion of patients eligible for UCB distribution who were provided with UCB.

### Data extraction and processing

Automatic hospital registration systems provided the numbers of contrast material-enhanced CT and MRI scans, administered CM type and volume (total daily volumes of intravenously administered CM, which were subsequently manually converted to grams by multiplying by the CM concentration used), patient status (hospitalized or outpatient), and patient postal codes of residence.

### Water sampling and chemical analysis

Flow-corrected continuous sampling was done by automated systems present at the WWTPs (Table 1). Daily flow-corrected samples of influent were those collected from 00:00 to 23:59 h at the WWTPs receiving effluents from participating centers. Samples were stored pending CM-concentration measurements according to a validated protocol [23]. Daily CM load in WWTP influent was the total daily volume entering the WWTP multiplied by the CM concentration in 24-h composite samples.

Certified chemical laboratories analyzed the samples using customized liquid chromatography with tandem mass spectrometry (LC-MS/MS) for iodine-based CM-molecules (iopromide and ioversol), and inductively coupled plasma mass spectrometry (ICP-MS) for gadolinium. Limits of quantification were 0.05 µg/L iopromide, 0.01 µg/L gadolinium for gadobutrol (Aqualysis Waterlaboratory), 0.1 µg/L ioversol, and 0.005 µg/L gadolinium for gadoteric acid (DVGW - TechnologieZentrum Wasser).

### Statistics

Categorical data are presented as absolute numbers and percentages. Differences in proportions between groups were tested using the Chi-square test. Distribution of CM concentrations in wastewater samples was compared using the nonparametric Mann–Whitney U test. Control- and intervention periods were compared for CM load in WWTP influent using linear regression with one-way analyses of covariance (ANCOVA), adjusted for the amount of intravenously administered CM. In order to account for the time elapsing between contrast material administration, hospital effluents reaching WWTP influent, and the start of WWTP sampling day, daily amounts of intravenously administered CM were paired with daily CM load in WWTP influent with a 24-h delay. Analyses were performed using IBM SPSS Statistics for Macintosh (version 29; IBM Corp). *P* values less than 0.05 were considered to indicate statistical significance.

## Results

**Fig. 2:**
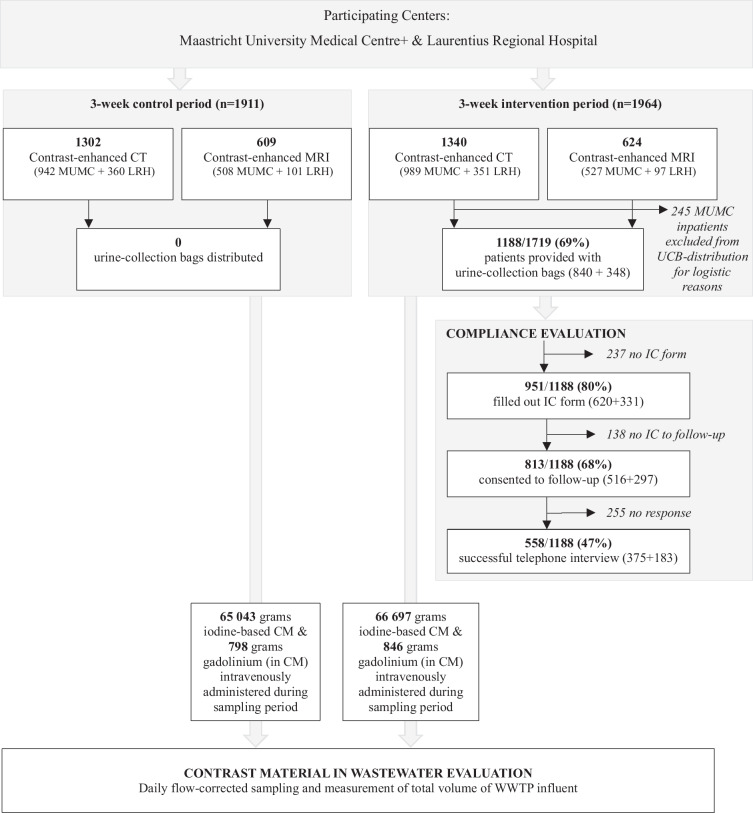
Study inclusion profile. MUMC, Maastricht University Medical Centre; LRH, Laurentius Regional Hospital; CT, computed tomography; MRI, magnetic resonance imaging; UCB, urine-collection bag; IC, informed consent; CM, contrast material; WWTP, wastewater treatment plant

An overview of the study inclusion profile is given in Fig. 2: 1911 contrast material-enhanced scans were performed during the control periods, and 1964 during the intervention period. Of the latter, 245 hospitalized patients (17% of eligible Maastricht UMC+ patients) were excluded from UCB distribution at Maastricht UMC+. During the two intervention periods, UCB were distributed to 69.1% (1188/1719) eligible patients.

### Control- and intervention period characteristics

Characteristics of control- and intervention periods are shown in Table 2: to ensure inclusion of the same days of the week, the effect of UCB distribution on CM load was evaluated during 2 × 20 days at Maastricht UMC+ and 2 × 19 days at Laurentius RH. Corresponding control- and intervention periods did not statistically differ in proportions of patients hospitalized or residing within local WWTP catchment areas (Appendix S1 shows CM-dispersion heat maps).

**Table 2:** Baseline characteristics of control and intervention periods at both participating centers

	Control period	Intervention period	*p*-value
**A. Maastricht University Medical Centre**			
First study day	Wednesday, 23 Nov 2022	Wednesday, 18 Jan 2023	
Last study day	Monday, 12 Dec 2022	Monday, 6 Feb 2023	
Duration	20 days	20 days	
Patients receiving iodine-based CM hospitalized or residing within the catchment area^†^	275/900 (30.6%)	267/919 (29.1%)	0.485
Patients receiving gadolinium-based CM hospitalized or residing within the catchment area^†^	102/484 (21.1%)	96/500 (19.2%)	0.458
Iodine-based CM administered (iopromide)	48,042 g	48,936 g	
Gadolinium administered (in gadolinium-based CM: gadobutrol)	684 g	734 g	
First water sampling day	Thursday, 24 Nov 2022	Thursday, 19 Jan 2023	
Last water sampling day	Tuesday, 13 Dec 2022	Tuesday, 7 Feb 2023	
Range of contrast material concentrations in WWTP influent samples*			
Iopromide (µg/L)	170 (115) range 253 (47–300)	105 (58) range 151 (29–180)	**0.014**
Gadolinium (µg/L)	0.95 (0.71) range 1.18 (0.42–1.60)	0.66 (0.43) range 0.94 (0.26–1.20)	**0.015**
**B. Laurentius Regional Hospital**			
First study day	Friday, 15 Mar 2024	Friday, 23 Feb 2024	
Last study day	Tuesday, 2 Apr 2024	Tuesday, 12 Mar 2024	
Duration	19 days	19 days	
Patients receiving iodine-based CM hospitalized or residing within the catchment area^†^	237/301 (78.7%)	229/311 (73.6%)	0.459
Patients receiving gadolinium-based CM hospitalized or residing within the catchment area^†^	66/86 (76.7%)	64/89 (71.9%)	0.469
Iodine-based CM administered (ioversol)	17,001 g	17,761 g	
Gadolinium administered (in gadolinium-based CM: gadoteric acid)	114 g	112 g	
First water sampling day	Saturday, 16 Mar 2024	Saturday, 24 Feb 2024	
Last water sampling day	Wednesday, 3 Apr 2024	Wednesday, 13 Mar 2024	
Range of contrast material concentrations in WWTP influent samples*			
Ioversol (µg/L)	23.5 (19.3) range 57 (11–68)	25.0 (14.0) range 77 (11–88)	0.869
Gadolinium (µg/L)	0.27 (0.21) range 0.42 (0.07–0.49)	0.21 (0.10) range 0.38 (0.04–0.42)	0.538

*CM* contrast material, *WWTP* wastewater treatment plant. *p*-values in bold type indicate statistical significance

^†^ Denominators indicate sample sizes, i.e. number of patients for which data on post-contrast hospitalisation status and place of residence was available. * Contrast material concentrations are reported as median (IQR), and are of the whole molecule for iopromide and ioversol, and of gadolinium for gadobutrol and gadoteric acid. Limits of quantification: iopromide 0.05 µg/L; gadolinium from gadovist 0.01 µg/L (Maastricht UMC+); ioversol 0.1 µg/L; 0.005 µg/L gadolinium from gadoteric acid (Laurentius RH)

All daily CM concentrations and total WWTP influent volumes could be reliably measured: minimum iodine-based CM and gadolinium concentrations were all above laboratory limits of quantification, and no hydraulic overloading occurred. Distributions of CM concentrations in wastewater samples were significantly different between corresponding control and intervention periods at Maastricht UMC+, giving a first inkling of successful CM load reduction. A similar difference was not seen at Laurentius RH (Table 2).

### Impact of urine-collection bag distribution

**Fig. 3:**
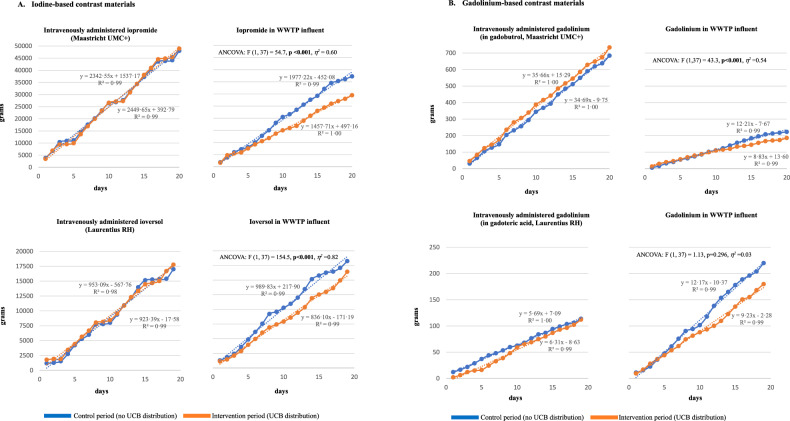
Daily cumulative amounts of **A**. iodine-based and **B**. gadolinium-based contrast materials intravenously administered and found in WWTP influent. WWTP, wastewater treatment plant; UMC, University Medical Center; RH, regional hospital; UCB, urine-collection bag. Segmental patterns in CM-administered plots reflect reduced CM administration during weekends. Except for gadolinium at Laurentius RH, UCB distribution during intervention periods had a statistically significant impact on CM load in WWTP influent, even after controlling for the amount of CM administered (ANCOVA). UCB distribution and the amount of CM administered almost fully accounted for variances in CM load in WWTP for these CM: iopromide *R*^2^ = 0.968 (adjusted *R* = 0.966); gadolinium Maastricht UMC+ *R*^2^ = 0.967 (adjusted *R*^2^ = 0.965); ioversol *R*^2^ = 0.990 (adjusted *R*^2^ = 0.989)

Day-to-day cumulative amounts of CM administered and in WWTP influent are depicted in Fig. 3. Plots of administered CM during control and intervention period closely align (segmental patterns reflect reductions during weekends). Plots of contrast material load in WWTP influent diverge toward less CM during the intervention period compared to the control period.

For all CM but gadolinium at Laurentius RH, linear regression analysis shows UCB distribution had a statistically significant impact on CM load, even after controlling for the amount of CM administered (Fig. 3): iopromide F(1,37) = 54.7, *p* < 0.001, *η*^2^ = 0.60; ioversol F(1,37) = 154.5, *p* < 0.001, *η*^2^ = 0.82; gadolinium Maastricht UMC+ F(1,37) = 43.3, *p* < 0.001, *η*^2^ = 0.54; gadolinium Laurentius RH F(1,37) = 1.13, *p* = 0.296, *η*^2^ = 0.03. UCB distribution and amount of CM administered almost fully accounted for variances in CM load in WWTP for these CM: iopromide *R*^2^ = 0.968 (adjusted *R*^2^ = 0.966); ioversol *R*^2^ = 0.990 (adjusted *R*^2^ = 0.989); gadolinium Maastricht UMC+ *R*^2^ = 0.967 (adjusted *R*^2^ = 0.965).

**Fig. 4:**
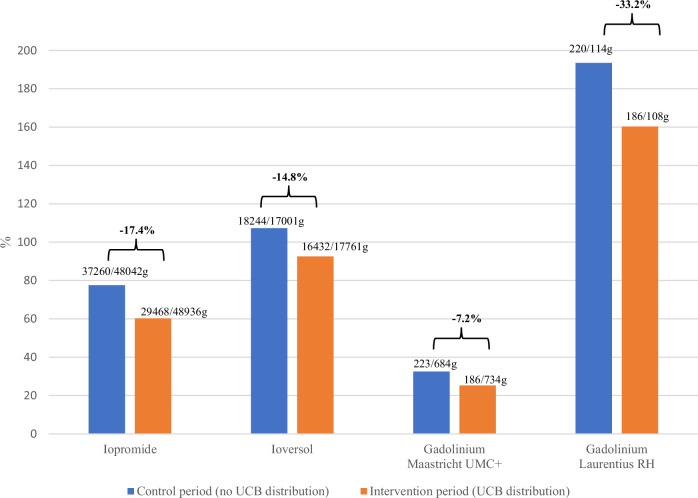
Proportions of intravenously administered contrast materials found in WWTP influent during control and intervention periods. UMC, University Medical Center; RH, regional hospital; UCB, urine-collection bag. Numbers represent total grams of contrast material intravenously administered/total grams of contrast material found in wastewater for each period. Gadolinium was administered in gadolinium-based contrast materials (gadobutrol at Maastricht UMC+ and gadoteric acid at Laurentius RH)

Figure 4 shows the proportion of intravenously administered CM found in WWTP influent during control and intervention periods. Proportions are consistently lower during intervention periods than during control periods (−17.4% iopromide; −14.8% ioversol; −7.2% Maastricht UMC+ gadolinium; and −33.2% Laurentius RH gadolinium). Except for ioversol during the intervention period, more CM was found in wastewater than was administered at Laurentius RH, reflecting CM “imported” into the local catchment area (absent for Maastricht UMC+; see Appendix S1).

### Compliance with using urine-collection bags

**Fig. 5:**
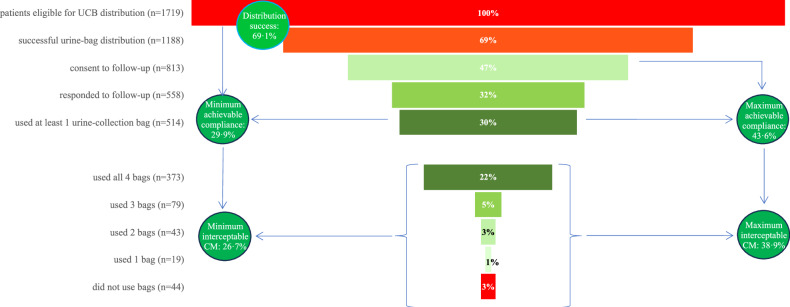
Urine-collection bag distribution in clinical practice: distribution success, compliance, number of urine-collection bags used, and achievable effects. Circles: Observed distribution successes was the proportion of patients eligible for UCB distribution that were actually provided with UCB. Minimum achievable compliance was defined as the proportion of patients eligible for UCB distribution (*n* = 1719) who confirmed using the UCB during the follow-up interview (*n* = 514). Minimum interceptable CM was defined as the minimum achievable compliance multiplied by the proportion of UCB used by interviewed and complying patients (*n* = 514 patients; 1834/2056 UCB, 89.2%). Maximum achievable compliance was defined as the proportion of patients eligible for UCB distribution (*n* = 1719) who consented to follow-up, irrespective of whether they were interviewed (*n* = 813, 47.3%), multiplied by known compliance, i.e., the proportion that used at least one UCB, of interviewed patients (514/558, 92.1%). Maximum interceptable CM was defined as maximum achievable compliance multiplied by the proportion of UCB used by interviewed and complying patients (89.2%, see above)

A funnel chart of overall UCB distribution and use is shown in Fig. 5: distribution success was 69.1% (1188/1719); 47.3% (813/1719) patients provided with UCB consented to follow-up; 47.0% (558/1188) consenting patients were successfully interviewed. Among interviewed patients, 92.1% (514/558) reported using at least one UCB, and together compliers used 89.2% (1834/2056) of UCB they were provided with. Data on where UCB were used were available for 1794/2056 (87.3%) of UCB distributed to complying interviewees: 119 (5.9%) were used at the hospital, 19 (1.1%) were used at work, and 1656 (92.3%) were used at home. Data on future UCB use was available for 517/558 (92.7%) interviewees: 473 (91.5%) stated they would use UCB in future.

**Fig. 6:**
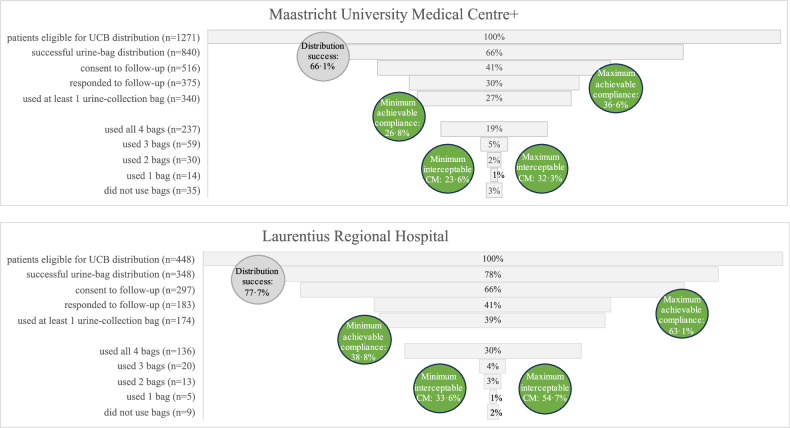
Urine-collection bag distribution in clinical practice at each participating center: distribution success, compliance, number of urine-collection bags used, and achievable effects. Circles: Observed distribution successes was the proportion of patients eligible for UCB distribution that were actually provided with UCB. Minimum achievable compliance was defined as the proportion of patients eligible for UCB distribution who confirmed using the UCB (Maastricht UMC+ 340/1271, 26.8%; Laurentius RH 174/448, 38.8%). Minimum interceptable CM was defined as minimum achievable compliance multiplied by the proportion of UCB used by interviewed and complying patients (Maastricht UMC+ 1199/1360 bags, 88.2%; Laurentius RH 635/732 bags, 86.7%). Maximum achievable compliance was defined as the proportion of patients eligible for UCB distribution who consented to follow-up (Maastricht UMC+ 516/1271, 40.6%; Laurentius RH 297/448, 66.3%), multiplied by known compliance of interviewed patients (Maastricht UMC+ 340/375, 90.1%; Laurentius RH 174/183, 95.1%). Maximum interceptable CM was defined as maximum achievable compliance multiplied by the proportion of bags used by interviewed and complying patients (Maastricht UMC+ 88.2%; Laurentius RH 86.7%)

Calculated minimum achievable compliance was 29.9% (514/1719), and minimum interceptable CM was 26.7% (29.9*0.89). Calculated maximum achievable compliance was 43.6% (813/1719*92.1), and maximum interceptable CM 38.9% (Fig. 5). The regional hospital may potentially achieve greater effects than the academic hospital (Fig. 6): Maastricht UMC+ 66.1% distribution success versus 77.7% at Laurentius RH; minimum achievable compliance 26.8% versus 38.8%; minimum interceptable CM 23.6% versus 33.6%; maximum achievable compliance 36.6% versus 63.1%; and maximum interceptable CM 32.3% versus 54.7%.

## Discussion

Overall UCB-distribution success during clinical practice was 69%. UCB distribution led to consistently lower CM loads in WWTP influent wastewater during intervention periods at both centers, with cumulative 3-week load reductions 15% and 17% for iodine-based CM and 7% and 33% for gadolinium. The impact of UCB distribution was statistically significant for iodine-based CM and gadolinium at the academic hospital. Except for the non-significant reduction in gadolinium at Laurentius RH, observed cumulative reductions were well below the compliance-based expected minimum (26.7%).

Compliance, 92% among interviewed patients, may have been overestimated. Although 68% of patients who were given UCB consented to a follow-up interview, only 47% (*n* = 558) were successfully interviewed. Current compliance data is based on the largest proportion of patients receiving UCB to date, and follow-up was in the form of personal interviews. Other studies on UCB use included 4% (*n* = 60) [24], 16% (*n* = 1523) [22], and 15–18% (*n* = 108) [20] patients in follow-up, which consisted of online/postcard questionnaires, and found 87%, 99% and > 92% compliance. A study published earlier this year evaluated compliance after contrast-enhanced CT scans in outpatients using telephone interviews: 503/671 (75%) eligible patients received UCB after their scan, 476 (95%) were successfully interviewed, and 455 (96%) reported having used at least one UCB [25]. In contrast to the current study, dedicated personnel were employed for UCB distribution and informed consent, which explains the higher rates of UCB distribution (75% versus 69%) and inclusion in telephone interviews (95% versus 47%). These results therefore reflect willingness to use UCB but not feasibility in clinical practice. It is important to note that the process from consent to response, to answers given during interviews, is susceptible to bias toward willing and complying patients, and self-reporting itself is subject to bias, leading to overestimation. Achievable results calculated in this study using compliance data therefore likely reflect upper limits.

Only one other study on UCB distribution evaluated corresponding changes in CM load: a reduction of 7% to 33% was found, which is in line with current results [24].

In the current study, the aim was to evaluate standard-care UCB distribution, but personnel motivation is expected to play an important part in distribution success. This is supported by the 75% distribution rate achieved by dedicated UCB-distribution personnel in the abovementioned study [25], as well as by the higher success rate found at Laurentius RH (78%), where 19 radiographers were involved in the study, compared to Maastricht UMC+ (66%), where ca. 120 radiographers are active. It may be that current results underestimate distribution success, and higher rates could be achieved through greater motivation. On the other hand, published distribution success rates to date are similar (60–70% and 75%), implying this may be representative of feasibility at this time [18, 24, 25].

To the best of our knowledge, this is the first study to evaluate the effect of UCB distribution through continuous CM-load monitoring at wastewater at WWTPs, measuring both iodine-based and gadolinium-based CM. In addition, the study provides an indication of differences between regional and academic hospital settings. Most importantly, UCB distribution was done using only the logistics and personnel already present for standard clinical practice, enabling evaluation of feasibility.

Quantifying CM load is fraught with confounders, not the least of which are external contributors to wastewater. This is reflected in the disparity of results between Maastricht UMC+ and Laurentius. Maastricht UMC+ administers high and consistent CM volumes and is in the unique position of being the main contributor to medical waste in local WWTP influent; CM import is the exception rather than the rule. Laurentius RH uses much less CM and does not administer any on some days. It is surrounded by a large population and catchment area, and the local WWTP receives three times as much wastewater, including wastewater from 4 other hospitals in the vicinity. These disparities explain why, despite the higher UCB-distribution success at Laurentius RH, CM loads exceeded administered amounts, less iodine-based CM reduction was achieved, and even relatively high differences in gadolinium (−33%) did not reach statistical significance. Furthermore, because data on clinically or environmentally relevant CM loads and effects of UCB distribution on CM loads are lacking, and as this is the first study of continuous CM-load monitoring, a formal power calculation could not be performed.

The current study did not register CM administered during procedures other than contrast material-enhanced CT or MRI. There are no other departments at either hospital administering gadolinium-based CM, but iodine-based CM is more widely used. For example, at Maastricht UMC+, seven other departments use iopromide and contribute toward iodine-based CM load. Similarly, whereas iodine-based CM load was determined based on the specific CM molecule, this was not possible for gadolinium-based CM, and the load was based on the element gadolinium. Gadolinium is almost entirely anthropogenic, geogenic levels are low, and the element is therefore eminently suited as a marker for pharmaceutical pollution [26]. However, in addition to its use in CM for MRI scans, gadolinium is utilized in nuclear reactors, alloys, as a phosphor in medical imaging, as a gamma ray emitter, in electronic devices, in optical devices, and in superconductors. Comparing periods neutralizes much of these confounders: corresponding control and intervention periods were in close proximity and included the same days of the week. There is no report of CM administration practices changing, and background ‘noise’ is not expected to differ between control and intervention periods to substantially affect results.

Cost-effectiveness studies should be done to investigate whether CM load reductions inferior to 20%, or even the estimated 39% maximum achievable, justify UCB distribution, or whether other strategies are preferable (collecting CM residues, prolonging in-hospital time to collect first post-CM urine, dedicated on-site filters, recycling, etc.) [17, 18, 27, 28]. Installing dedicated separation- or filter toilets may be beneficial, not only in removing CM from wastewater but also for recycling [27, 28]. However, installation and maintenance costs, as well as the required prolonged hospital stays, may be deterrents to widespread implementation. On the one hand, in the current study, 92% of UCB were used at home, and reported willingness to continue using UCB in future was high (92%). On the other hand, if all patients are given UCB, a substantial increase in healthcare costs will ensue, depending on local UCB prices and the number of contrast-enhanced scans. In the Netherlands, with an estimated 1,700,000 contrast-enhanced scans a year [1], distributing UCB (4 euro per set of 4) with 69% distribution success will translate to yearly recurring UCB acquisition costs of 4.7 million euro. In addition, there will be costs associated with (extra) hospital personnel, UCB distribution (information leaflets, etc.), and waste disposal (extra burden for retrieval and incineration of UCB).

Abovementioned costs and effectiveness aside, recent work has highlighted the significant contribution of radiology departments to healthcare-related energy consumption and carbon emissions, emphasizing the need for comprehensive evaluations of both direct and indirect environmental costs when implementing mitigation strategies such as UCB distribution [29]. Considering the huge number of UCB that would be required to intercept the hundreds of tons of CM administered annually worldwide, UCB production and disposal as non-recyclable waste, which is generally incinerated, will generate its own pollution as well as a high carbon footprint [1, 21], and will furthermore not allow recovery or recycling. Exact magnitudes of environmental costs are difficult to quantify, since pollution and carbon footprint are dependent on many factors, including UCB type and local distribution and waste trajectories. However, tentative worldwide estimates can be made based on the detailed evaluation of the environmental impact of 5 types of UCB from production to incineration by Tauw [21], together with the estimated number of contrast-enhanced CT and MRI scans performed worldwide (120 million and 40 million a year, respectively) [1]. If 4 UCB were distributed after each contrast-enhanced CT and MRI scan worldwide, projected annual increases could amount to 20–29 kilotons extra waste, 64–109 kilotons extra carbon dioxide, and an extra 6–11 million euro in costs to restore incurred environmental damage (as per the environmental cost indicator, ECI) [30]. Upon widespread implementation, another aspect to contend with will be littering, i.e., UCB being discarded in outdoor environments, which may become a substantial problem as was seen for facemasks in recent years [31].

Reduction in wastewater CM load may be achieved at less monetary and environmental cost by collecting first post-scan urine on-site [2]. Indeed, a recent study found that 92.7% (422/455) patients agreed to a prolonged hospital stay to collect first post-contrast material urine in dedicated canisters, which led to median recovery of 51.2% iodine-based and 12.9% gadolinium-based CM [32]. Limits in methodology mean these recovery rates may have been overestimated, but even so, these results are promising.

Effects of UCB distribution, represented by a divergence between cumulative CM load during control and intervention periods, were seen with a three to 11-day delay. This is in line with previous research, and probably reflects not only the requirement of larger cumulative CM amounts for an effect to become visible, but also differential behavior of iodine- and gadolinium-based CM in wastewater systems [33–35]. Although the current data-collection periods sufficed to detect differences in CM load, it is not known whether larger effects may become visible after sustained UCB distribution and wastewater sampling.

The exclusion of hospitalized patients at Maastricht UMC+ from UCB distribution may have led to lower effects on CM load in local WWTP influent. However, using compliance-based estimates, maximum CM load reductions for these hospitalized patients would be an additional 5.5%, bringing total reductions to levels that are still below the compliance-based expected minimum.

No data was collected on reasons for compliance or using a certain number of UCB. Similarly, registration of when UCB was used, how long (hospitalized) patients remained at the hospital after their scan, or how much CM was intercepted in UCB were beyond the current scope. The diuretic properties of (iodine-based) CM may stimulate urination [36], but it is not known whether this is so, what the delay is between scan and first post-scan urine, or how much CM is eliminated in this first post-scan urine. Such data is crucial for refining and optimizing UCB distribution or to develop alternative, more effective mitigation strategies, and should be the focus of future studies.

## Conclusion

UCB distribution had a significant but small impact on reducing wastewater CM loads. Current results show that compliance data overestimate UCB-distribution impact, and underscore the importance of wastewater measurements when evaluating mitigation strategies. CM interception at the source, asking patients to remain at the hospital until after their first urination, for example, may yield better and more certain results.

## Supplementary information

ELECTRONIC SUPPLEMENTARY MATERIAL

## Abbreviations

CM: Contrast material
CT: Computed tomography
MRI: Magnetic resonance imaging
RH: Regional hospital
UCB: Urine-collection bag
UMC: University Medical Center
WWTP: Wastewater treatment plant
