# Supplementary material for: Effect of distributing urine-collection bags on contrast-material load in wastewater
Source: Eur Radiol. 2025 Oct 17;36(3):2353–66. doi: 10.1007/s00330-025-11984-5 (PMC12963271; doi:10.1007/s00330-025-11984-5)
Supplement: Supplementary file 1 — ELECTRONIC SUPPLEMENTARY MATERIAL [file 330_2025_11984_MOESM1_ESM.pdf]

Effect of distributing urine-collection bags on contrast-material load in wastewater

ELECTRONIC SUPPLEMENTARY MATERIAL

Eur Radiol (2025) Janssen BJA, Zijta FM, Fraters N, et al

Appendix S1. Patient provenance and contrast material dispersion

Proportions of patients residing within the same catchment area as the hospital were: Maastricht UMC+ iopromide 30%, Laurentius RH ioversol 75%, Maastricht UMC+ gadobutrol 20%, and Laurentius RH gadoteric acid 74%.

Note that 5·8% of Maastricht UMC+ patients resided within the Laurentius RH WWTP catchment area, whereas no Laurentius RH patients resided within the Maastricht UMC+ WWTP catchment area.

WWTP = wastewater treatment plant. MUMC = Maastricht University Medical Center. LZR = Laurentius Regional Hospital.

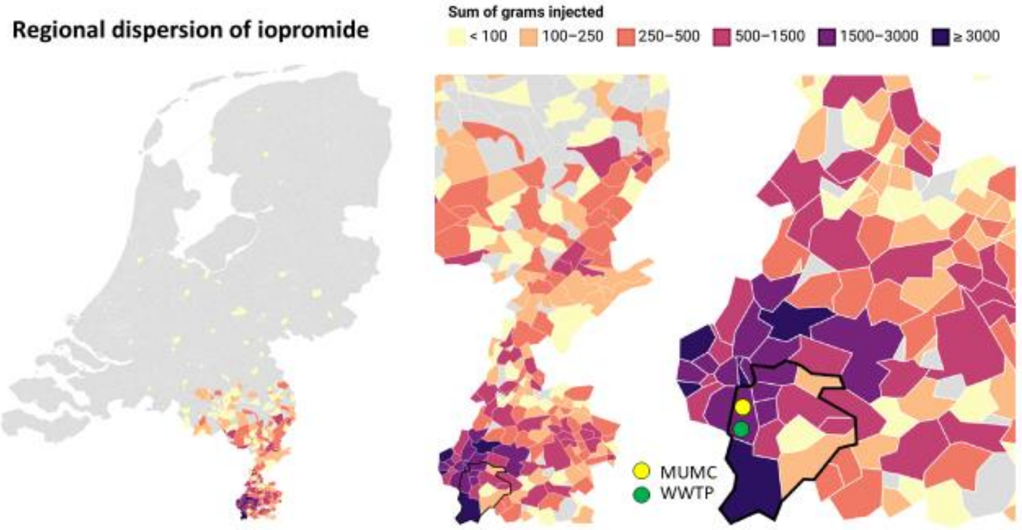

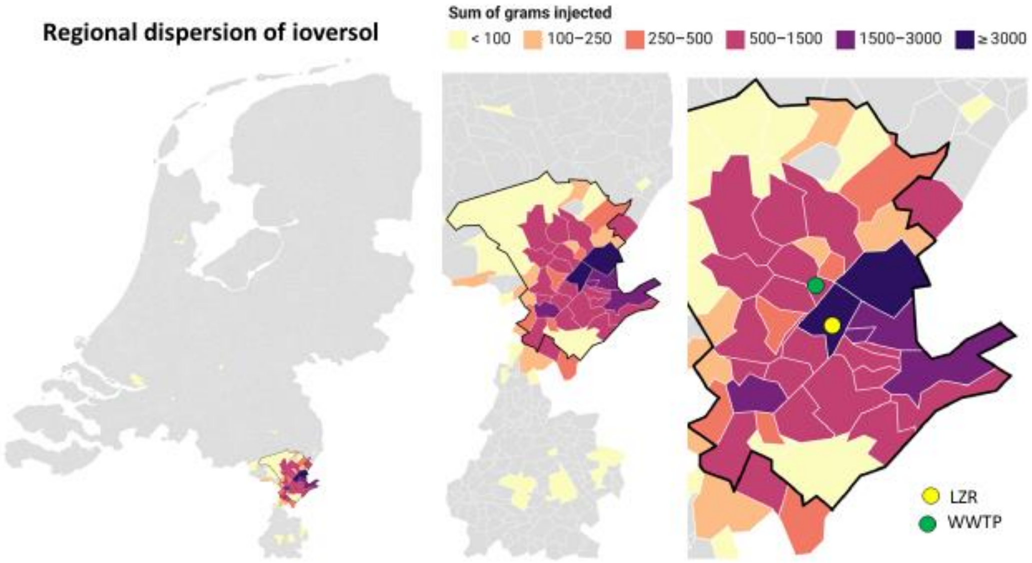

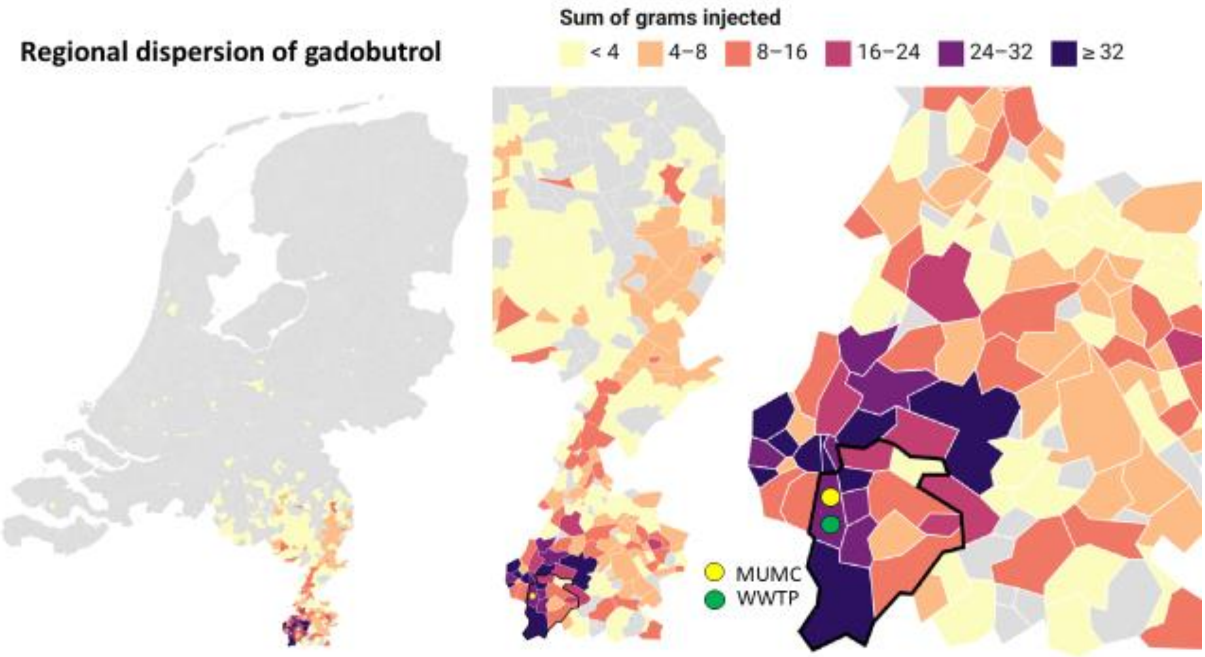
Regional dispersion of gadobutrol
Sum of grams injected
< 4
4–8
8–16
16–24
24–32
≥ 32
MUMC
WWTP

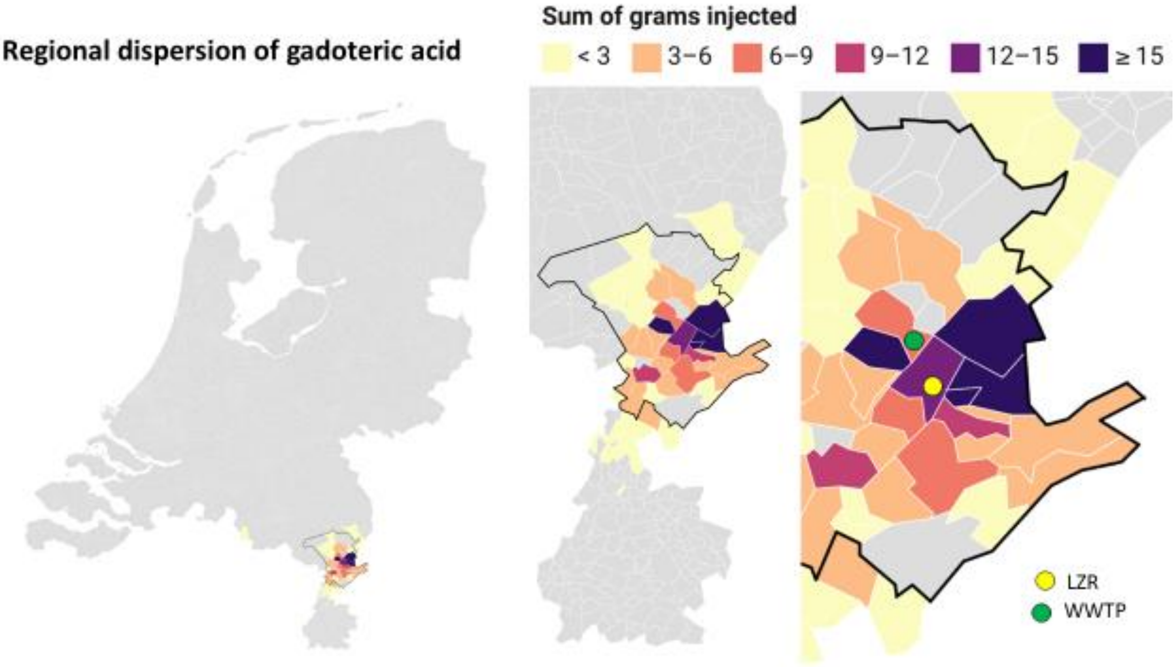
Regional dispersion of gadoteric acid
Sum of grams injected
< 3
3–6
6–9
9–12
12–15
≥ 15
LZR
WWTP
